# Zero Discharge of Nutrient Solution to the Environment in a Soilless Greenhouse Cucumber Production System

**DOI:** 10.3390/plants11172252

**Published:** 2022-08-30

**Authors:** Xiaotao Ding, Lizhong He, Rongguang Li, Tingting Qian, Hongmei Zhang, Haijun Jin, Jiawei Cui, Hong Wang, Qiang Zhou, Jun Zou, Dafeng Hui, Yuping Jiang, Kun He, Jizhu Yu

**Affiliations:** 1 Shanghai Key Lab of Protected Horticultural Technology, Horticultural Research Institute, Shanghai Academy of Agricultural Sciences, Shanghai 201403, China; 2 Shanghai Institute of Technology, College of Ecological Technology and Engineering, Shanghai 201418, China; 3 Shanghai Institute of Technology, College of Sciences, Shanghai 201418, China; 4 Department of Biological Sciences, Tennessee State University, Nashville, TN 37209, USA

**Keywords:** cucumber, zero discharge, growth, fruit quality, yield, greenhouse

## Abstract

With the development of the economy and society, more attention is being paid to energy costs and the potential environmental pollution caused by vegetable cultivation. The aim of this study was to investigate the impacts of zero discharge of nutrient solutions on cucumber growth, leaf photosynthesis, and the yield and quality of cucumber under greenhouse conditions. The results show that zero discharge treatment did not change plant height, stem diameter, internode length, leaf area, net photosynthetic rate (*P*_n_), stomatal conductance (*G*_s_), intercellular CO_2_ concentration (*C*_i_), transpiration rate (*T*_r_), and leaf relative chlorophyll content on the most measurement days. Only *P*_n_ and relative chlorophyll content were significantly reduced after 16 days of treatment but soon recovered over time. Cucumber plants can adapt to treatment circumstances over the course of days. Leaf mineral element contents showed significant differences on some treatment days compared to the control, and trace elements of Fe, Mn, Cu, and Mo can be appropriately supplied during the treatment days. The cucumber yield and fruit quality in the zero discharge treatment did not change during the whole experimental period. This study confirmed that the irrigation method of a nutrient solution with zero discharge can be applied in cucumber cultivation practices. The strict management of irrigation strategy, plant growth, and greenhouse climate are very important for zero discharge cultivation. The cultivation method with zero discharge of nutrient solution can reduce the energy costs of disinfection, save water and fertilizers, and reduce the environmental pollution in cucumber cultivation.

## 1. Introduction

Soilless cultivation or hydroponics is a method of growing plants without using soil as a rooting medium and plays a significant role in supporting efficient and intensive plant production [1]. To sustain the rapidly increasing global population, it is very important to increase horticultural production around the world, while reducing adverse impacts on the environment by improving resource utilization efficiency regarding water and nutrients [2,3].

The management of nutrient solutions is a vital component in soilless cultivation. Irrigation strategy mainly depends on radiation accumulation, and the drainage of a nutrient solution, which reaches 20–30% of the daily applied irrigation, is controlled by a computer in modern greenhouses [4]. The reuse of drainage is not only due to the restriction of environmental laws and regulations that aim to reduce the nutrient leaching to the environment, but also for significant savings of water and nutrients. Normally, the drainage water is disinfected by heating [5], ultraviolet (UV) irradiation [6,7], or chemical treatments of hydrogen peroxide (H_2_O_2_) [8], ozone (O_3_) [9], and chlorine [10]. Whatever the disinfection methods used, there are a series of drawbacks, such as the high cost of equipment, high energy consumption, reactions with fertilizers and organics, and potentially toxic by-products [11]. Furthermore, over 10% of discharges could potentially be discharged because of a calamity in the disinfection equipment [12].

Coconut coir is a waste product of the coconut industry, consisting of the dust and short fibers derived from the mesocarp of the fruit. Coconut coir is widely used in greenhouse vegetable cultivation [1,13]. Rockwool is commonly used in greenhouses in the Netherlands. As there are only a few companies that can produce and retrieve rockwool, coconut coir is more commonly used in soilless cultivation in China. Cucumber (*Cucumis sativus* L.) is one of the most popular vegetables cultivated in greenhouses worldwide. There are many studies that focus on cucumber growth, development, physiology, and yield in greenhouses [14], but the studies about recirculating nutrient solutions with zero discharge under coir cultivation of cucumber are rare.

The purpose of the study was to explore the effects of zero discharge of nutrient solution on cucumber growth, yield and fruit quality under coir cultivation in a greenhouse, so as to find an easy method for fully utilizing nutrient water without affecting the yield and quality of cucumbers in greenhouses. In this study, we carried out two treatments, including normal irrigation and zero discharge irrigation, for cucumbers in a Venlo-type greenhouse in Shanghai and investigated the effects of different irrigation methods on plant growth, leaf photosynthesis, and the yield and fruit quality of cucumbers. 

## 2. Results

### 2.1. Changes in Environmental Parameters outside and inside the Greenhouse

**Figure 1:**
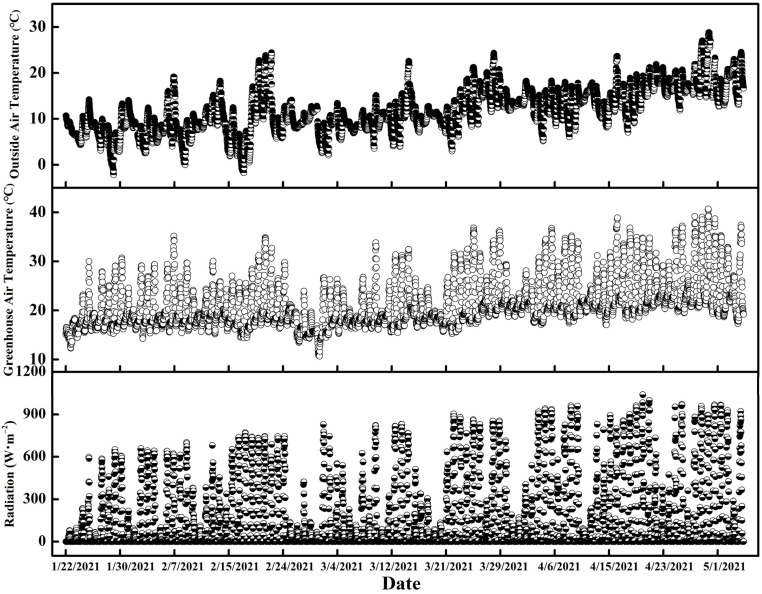
Changes in greenhouse air temperature, radiation and outside air temperature between 22 January and 5 May 2021. Data of greenhouse air temperature and outside air temperature were recorded every half an hour.

The outside air temperature, radiation and greenhouse air temperature gradually increased from January to May (Figure 1). The maximum outside air temperature and greenhouse air temperature were 28.8 °C and 40.7 °C, the minimum temperatures were −2.2 °C and 10.7 °C, and the average temperatures were 11.6 °C and 21.2 °C, respectively. The maximum radiation was 1039 W·m^−2^. During the experimental period from 22 January to 5 May 2021, these maximum parameters appeared in April, when the radiation and greenhouse air temperature were both at high levels, especially during the middle and toward the end of April.

### 2.2. Changes in Plant Height, Stem Diameter, Internode Length, and Leaf Area per Plant

**Figure 2:**
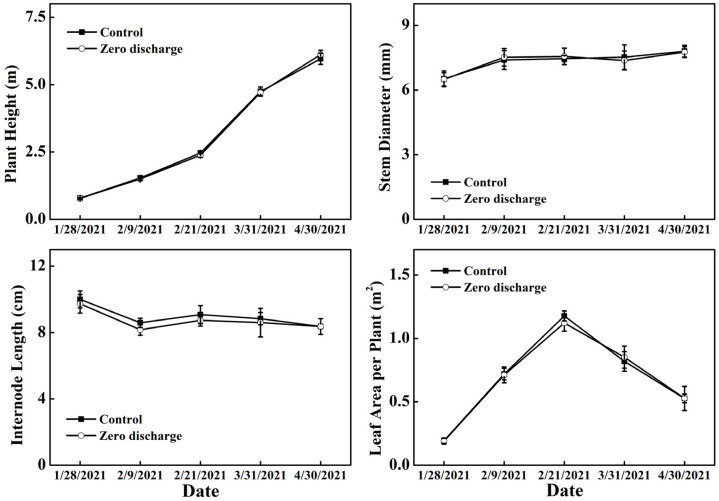
Changes in plant height, stem diameter, internode length, and leaf area in different treatments 8 d before treatment and 4 d, 16 d, 54 d, and 84 d after treatment. Control and zero discharge represent normal irrigation and zero discharge irrigation. Data are means of at least three biological replications.

There were no significant differences in plant height, stem diameter, internode length, or leaf area between the control (normal irrigation) and zero discharge treatments at different treatment times (Figure 2). For both zero discharge of nutrient solution and normal irrigation treatments, the plant height increased in parallel with the treatment days, the stem diameter and internode length did not significantly change with time, and the highest leaf area was found 16 d after treatment. The leaf area decreased after 16 days of treatments.

### 2.3. Changes in Leaf Gas Exchange Parameters, and Relative Chlorophyll Content

**Figure 3:**
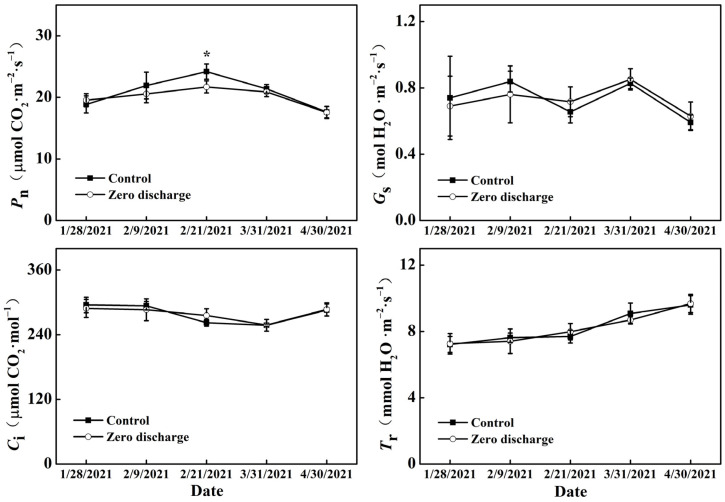
Changes in cucumber net photosynthetic rate (*P*_n_), stomatal conductance (*G*_s_), intercellular CO_2_ concentration (*C*_i_) and transpiration rate (*T*_r_) of different treatments 8 d before treatment and 4 d, 16 d, 54 d, and 84 d after treatment. Control and zero discharge represent normal irrigation and zero discharge irrigation. The data are means of at least three biological replications. “*” indicate significant differences at α = 0.05 based on the least significant difference (LSD) test.

The leaf net photosynthetic rate (*P*_n_), stomatal conductance (*G*_s_), intercellular CO_2_ concentration (*C*_i_), and transpiration rate (*T*_r_) were not significantly influenced by the different irrigation treatments (Figure 3) for all days except for the *P*_n_ of zero discharge treatment, which was significantly lower for the zero discharge treatment than the control after 16 d of treatment. The *T*_r_ gradually increased in parallel with the treatment days.

**Figure 4:**
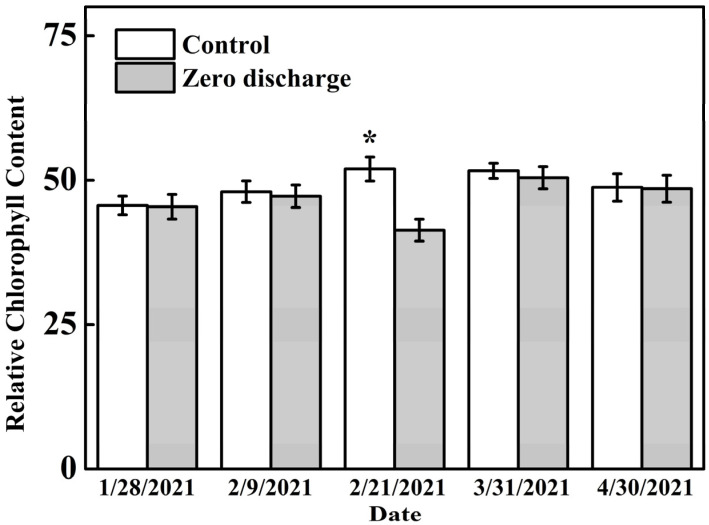
Changes in the relative chlorophyll content of different treatments 8 d before treatment and 4 d, 16 d, 54 d, and 84 d after treatment. Control and zero discharge represent normal irrigation and zero discharge irrigation, respectively. Data are means of at least five biological replications. “*” indicate significant differences at α = 0.05 based on the least significant difference (LSD) test.

There were no significant differences in relative chlorophyll content between the control and zero discharge treatments 8 d before treatment, or 4 d, 54 d, and 84 d after treatment (Figure 4). Compared to the control, the zero discharge treatment significantly decreased the relative chlorophyll content 16 d after treatment.

### 2.4. Changes in Leaf Mineral Elements

**Figure 5:**
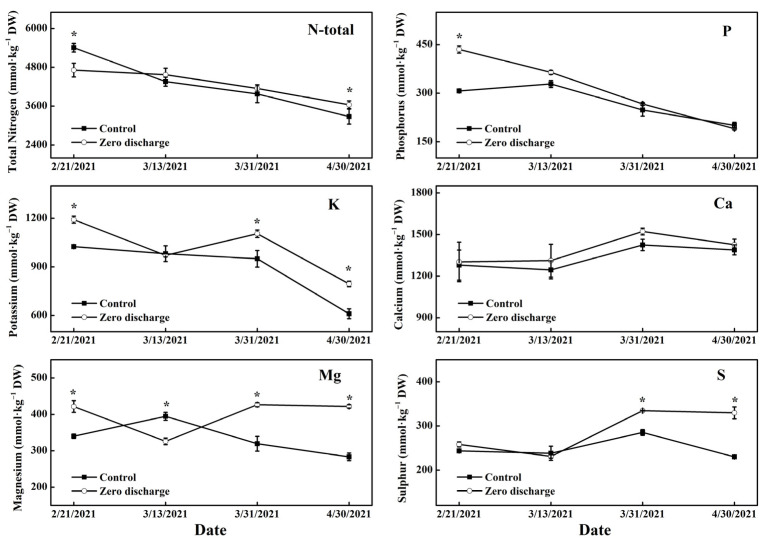
Changes in the leaf macro minerals content of different treatments after 16 d, 36 d, 54 d, and 84 d of treatment. Control and zero discharge represent normal irrigation and zero discharge irrigation. Data represent the mean ± SD (*n* = 3). “*” indicates significant differences at α = 0.05 based on the least significant difference (LSD) test.

Compared to the control, the leaf total N content of the zero discharge treatment significantly decreased after 16 d of treatment, and there were no significant differences between them after 36 d and 54 d of treatment, while the leaf total N content of the zero discharge treatment significantly increased after 84 d of treatment (Figure 5). The leaf P content of the zero discharge treatment was clearly higher than the control after 16 d of treatment, and there were no significant differences on the following treatment days. Leaf K content of the zero discharge treatment was significantly increased compared to the control after 16 d, 54 d, and 84 d of treatment, and there was no significant difference after 36 d of treatment. For the Ca content, there were no significant differences between the control and zero discharge for all the treatment days. The leaf Mg content of the zero discharge treatment significantly increased compared to the control after 16 d, 54 d and 84 d of treatment and was significantly decreased after 36 d of treatment. Compared to the control, there was no significant difference between the leaf S content of zero discharge after 16 d and 36 d of treatment, but this significantly increased after 54 d and 84 d of treatment.

**Figure 6:**
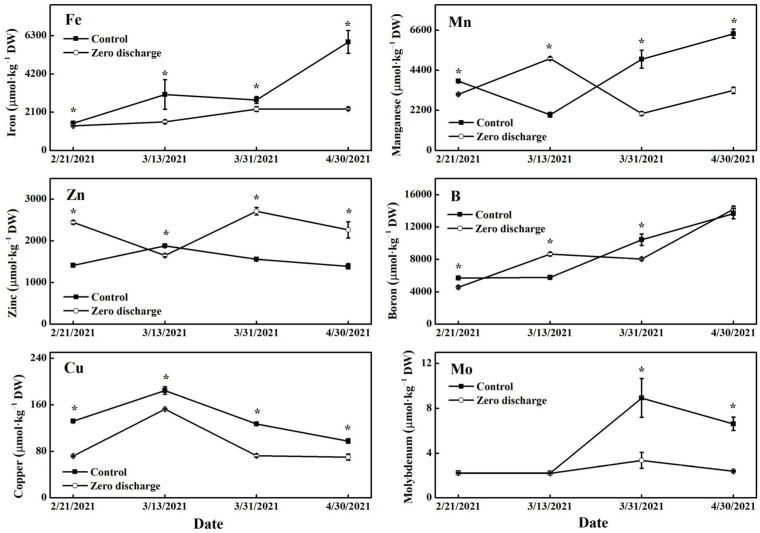
Changes in leaf trace elements content of different treatments after 16 d, 36 d, 54 d, and 84 d of treatment. Control and zero discharge represent normal irrigation and zero discharge irrigation. Data represent the mean ± SD (*n* = 3). “*” indicate significant differences at α = 0.05 based on the least significant difference (LSD) test.

For different treatment times, leaf Fe and Cu contents of the zero discharge treatment were both significantly decreased compared to the control (Figure 6). Leaf Mn content of the zero discharge treatment was lower than the control after 16 d, 54 d, and 84 d of treatment, and was higher than the control after 36 d of treatment. The reverse changes in leaf Zn content were found in the treatments compared with Mn content. The leaf B content of the zero discharge treatment was significantly decreased after 16 d and 54 d of treatment compared to the control and was significantly increased after 36 d of treatment. There was a significant decrease in leaf Mo content of the zero discharge treatment compared to the control after 54 d and 84 d of treatment.

The averaged leaf macro minerals of P, K, Mg, and S were significantly increased in the zero discharge treatment compared to the control, and the other macro minerals N and Ca showed no significant difference between the zero discharge and control treatments (Table 1). The averaged leaf trace elements of Fe, Mn, Cu, and Mo were significantly decreased, while Zn was significantly increased in the zero discharge treatment.

### 2.5. Changes in Cucumber Fruit Quality and Yield

**Figure 7:**
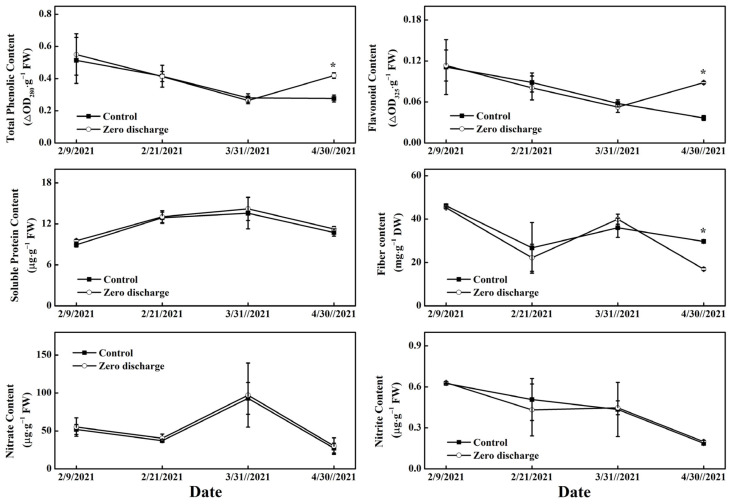
Changes in cucumber fruit quality parameters of different irrigation treatments after 4 d, 16 d, 54 d, and 84 d of treatment. Control and zero discharge represent normal irrigation and zero discharge irrigation, respectively. Data represent the mean ± SD (*n* = 3). “*” indicates significant differences at α = 0.05 based on the least significant difference (LSD) test.

The measured cucumber fruit quality included total phenolic, flavonoid, solute protein, fiber, nitrate and nitrite contents (Figure 7). Compared to the control, the total phenolic and flavonoid contents of fruit for the zero discharge treatment were significantly increased after 84 d of treatment, while lower fiber content was found at the time. There were no significant differences in solute protein, nitrate, and nitrite contents between the control and zero discharge treatments for all of the treatment times.

**Figure 8:**
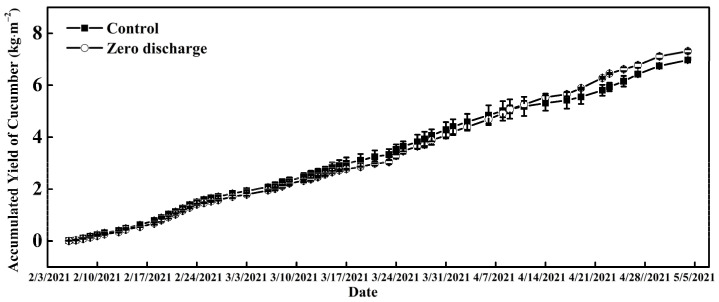
Changes in the accumulated yield of cucumber of different treatments. Control and zero discharge represent normal irrigation and zero discharge irrigation, respectively. Data represent the mean ± SD (*n* = 3).

The accumulated yield of cucumber gradually increased over time for both the control and zero discharge treatments, and the value of the zero discharge treatment was a little higher than the control at the end of all harvest times (Figure 8). There was no significant difference in yield per area between them.

## 3. Discussion

Irrigation is an important management practice that provides water, nutrients, and oxygen to plants in order to satisfy their needs for growth and production. In this study, we compared zero discharge irrigation and normal irrigation treatments and their effects on the growth, yield and fruit quality of cucumber in a soilless greenhouse production system. The results show that there were no significant differences in plant height, stem diameter, internode length, leaf area, *G*_s_, *C*_i_, and *T*_r_ between the control and zero discharge treatments; only the *P*_n_ and relative chlorophyll content in the zero discharge treatment exhibited a significant decrease after 16 days of treatment. As the changes in gas exchange parameters and chlorophyll content are directly related to plant health and growth conditions [15,16], our results indicate that the zero discharge treatment had a limited adverse influence on cucumber plants. Cucumber was not immediately influenced by the zero discharge treatment, and only 16 days after treatment, the cucumber leaves showed significantly low chlorophyll content and *P*_n_, but soon recovered over time. The phenomena of this situation also illustrated that the zero irrigation treatment was adequate for cucumber production, and cucumber plants could adapt to the treatment circumstance over days [17].

Nutrients mainly enter plants via their roots, with the relevant nutrients dissolved in water. Nevertheless, some nutrients, especially trace elements, such as iron, zinc, copper, and manganese, are often applied via foliar spray [1]. In our study, 16 days after the zero discharge treatment, leaf N, Fe, Mn, B, and Cu were significantly decreased compared to the control. A pale green color was found for leaves in the zero discharge treatment, while simultaneously, a dark green color was found in the control. Therefore, the foliar spraying of some nutrients can be performed in cultivation practice at the time of the experiment. The differences in elements between the control and zero discharge treatments became smaller with the treatment days, mainly because of the adaptation to the treatment. The reasons for discharge in growers’ traditional irrigation practices included concerns regarding a high sodium level, filter rinsing water, fear of diseases, and an imbalance in the circulating nutrient solution [12]. In our study, cucumber plants in the zero discharge treatment grew well during most of the cultivation times, and mineral contents in leaves were mainly in the health ranges according to Tsukagoshi et al. [18].

The fruit quality is important to growers and a key issue to be improved by the management of cucumber production [19,20]. Total flavonoids and phenols are closely related to antioxidants, which can prevent and suppress various diseases and greatly affect consumer preference for cucumber fruit [21,22], and soluble protein, fiber, nitrate, and nitrite contents are important quality parameters [23]. In this study, most of the quality parameters were not significantly different between the control and zero discharge treatments; only in the last measurement were some differences found. These illustrated that the zero discharge treatment had no negative effect on cucumber fruit quality. The reason for the difference in the last measurement was mainly due to plant senescence caused by high greenhouse temperatures and the invasion of red spiders. Research has been conducted to decrease the accumulation of nitrate and nitrite [24,25]. In our study, we were also concerned about the potential increases in nitrate and nitrite content in the zero discharge treatment. To our pleasant surprise, the contents of nitrate and nitrite in the zero discharge treatment were mostly maintained at the control level.

**Figure 9:**
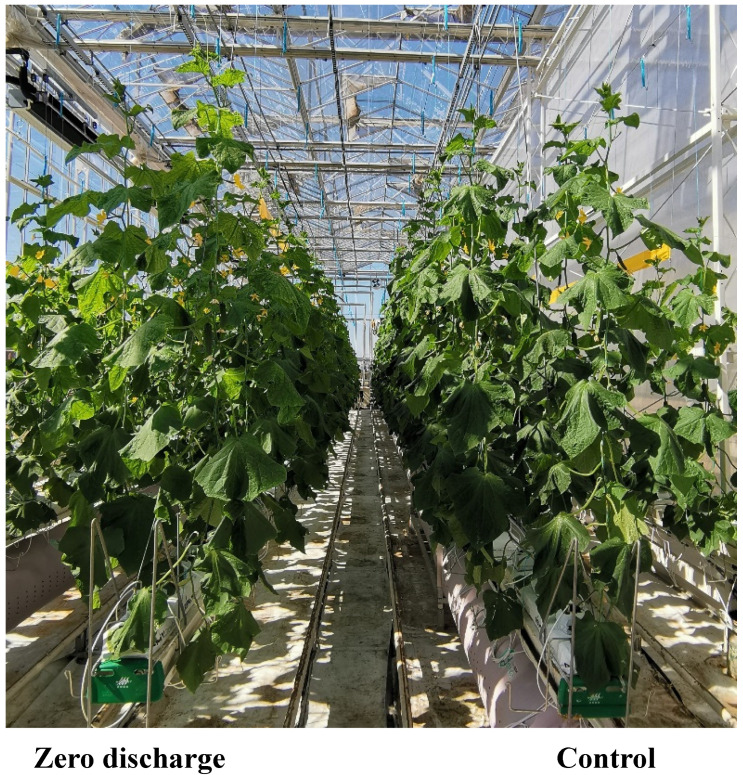
Growth picture of cucumber plants in the greenhouse.

**Figure 10:**
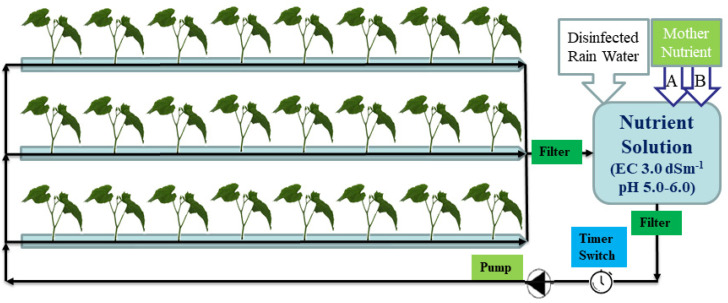
Schematic overview of the zero discharge irrigation system.

The cucumber yield was not significantly influenced by the zero discharge treatment in the experiment, confirming that the method can be applied in cucumber cultivation practice in greenhouses in spring or winter (Figure 9). Root diseases were not observed in the experiment, which may be due to the clean irrigation water and substrate, and the relatively low temperature at the beginning of the experiment. The high greenhouse temperature in the middle and end of April led to plant senescence, and red spiders emerged, spreading quickly and suppressing cucumber yield. Thus, we stopped cucumber cultivation in early May. We propose a schematic system of zero discharge of nutrient solution that can be used in cucumber cultivation practices (Figure 10). The strict management of irrigation strategy, plant growth, and greenhouse climate is very important in the zero discharge of nutrient solution cultivation.

## 4. Materials and Methods

The experiments were conducted in a semi-closed Venlo-type glass greenhouse at the Chongming base of the National Engineering Research Center of Protected Agriculture (31°34′ N, 121°41′ E), Shanghai Academy of Agriculture Sciences (Shanghai, China) in 2021. The cucumber variety used in this study was Deltastar (Rijk Zwaan Company, De Lier, Netherlands). The cucumber grew under natural light in a well-heated greenhouse of about 280 m^2^. Greenhouse CO_2_ was supplied by natural ventilation and air treatment unit (ATU) air circulation during the cucumber growth. For a more detailed description of the climate control, irrigation strategy and plant management, please refer to Ding et al. [4].

Outside temperature, radiation, and air temperature in the greenhouse were automatically monitored. Measurements were automatically recorded at 30 min intervals using climate sensors (Priva, De Lier, The Netherlands) in the greenhouse.

The substrate used in the experiment was coconut coir (EC < 1, pH 5.8–6.8; Qingdao Remmy Commerce and Trade Co., Ltd., Qingdao, China). A block with 100% coconut coir granules (0–6 mm) about 10 cm × 10 cm × 6.5 cm was used for cucumber seedling growth, and a slab with coconut coir granules (50% of 0–6 mm, and 50% of 10–20 mm) about 100 cm × 20 cm × 8 cm was used for cucumber planting. The raw water used for irrigation came from rainwater, after the colloidal and suspended particles, fungi, proteins, bacteria, viruses, etc., were removed by ultrafiltration [11]. Before sowing and planting, the coconut coir blocks and slabs were soaked in nutrient solution (EC 3.0 dS·m^−1^, pH 5.5) for 24 h, and then the remaining solution was drained. The original mother nutrient solutions (A and B) are listed in Table 2. Every time the same amounts of A and B were used by the fertilizer applicator, the EC was controlled by a computer. Nitric acid (HNO_3_) and sodium hydroxide (NaOH) were used to adjust the pH of the nutrient solution. We grew the cucumber seedlings until the sixth true leaf had expanded and transplanted them into the greenhouse (22 January 2021). Cucumber seedlings were irrigated by drip fertigation controlled by a computer, for which the drain nutrient solution was about 20–30% of the applied after two weeks of planting. Plants were pruned to a single stem and one flower was kept at each node from the third node onwards. Stem density was 1.6 stems m^−2^, and plants were grown using a high-wire growing system. After two weeks of normal irrigation in the coconut coir slabs, the plant roots grew well, and we started zero discharge irrigation treatment (see description of treatments below). We ended the experiment on 5 May 2021, as the greenhouse temperature was, to some extent, too high for plant growth, the cucumber became senescent, and red spiders emerged and spread quickly.

The two irrigation treatments are designed as below: (1) Control: normal irrigation, controlled by the computer, irrigated nutrient solution (EC 3.0 dS·m^−1^, pH 5.0–6.0) from fertilizer applicator, with the drain nutrient solution reaching up to 20–30% of the applied every day [3,4], and all the drainage was temporarily collected in a big container. (2) Zero discharge: irrigation method of nutrient solution with zero discharge. Nutrient solution (EC 3.0 dS·m^−1^, pH 5.0–6.0) was irrigated by a pump (550 W) from container one (500 L), and the nutrient solution also came from the fertilizer applicator that was stored in container two (1000 L). After irrigation, the drainage floated into container one and mixed with clean rainwater (by ultrafiltration), and the new nutrient solution (which came from container two) every day to ensure that the irrigate nutrient solution was always EC 3.0 dS·m^−1^, pH 5.0–6.0 in the whole cultivation period. We used a block design with three replications. Each treatment block was set to about 40 m^2^ and repeated three times. From 5 February 2021 to 20 March 2021, we irrigated the plants in the zero discharge treatment six times every day, and the irrigation times were set as: 9:00–9:05, 11:00–11:06, 12:00–12:06, 13:00–13:06, 14:00–14:06, 15:00–15:03. From 21 March 2021, to the end of the experiment, we increased irrigation three to four times in the sunny days as the radiation clearly increased to make sure that there was enough irrigation for cucumber growth. The same irrigation strategy was applied to the control. The nutrient solution in container one was tested every day, to ensure that there was enough nutrient solution; the irrigation nutrient solution EC was 3.0 dS·m^−1^, pH 5.0–6.0, guaranteeing that the drain nutrient solution floated into container one every time.

### 4.1. Measurements of Cucumber Yield, Plant Height, Stem Diameter, Internode Length, and Leaf Aera

Normally, the cucumber fruits were harvested every 1–2 days, the yields were recorded every time, and the accumulation yield was calculated for future analysis. Cucumber plant height, stem diameter, internode length, leaf length, and leaf width were measured 8 days (28 January 2021) before treatment and 4 days (9 February 2021), 16 days (21 February 2021), 54 days (31 March 2021), and 84 days (30 April 2021) after treatment. Five plants were randomly selected for each measurement, and positions from about one meter to the top of the plant were selected for stem diameter and internode length measurements. The length and width of total leaves from the top to the bottom were measured with a caliper (Ding et al.) [14]. The leaf area was estimated according to the method of Cho et al. [26], in which the leaf area (SA) = −210.61 + 13.358 × leaf width + 0.5356 ×leaf length × leaf width.

### 4.2. Measurements of Outside Air Temperature, Radiation, and Greenhouse Air Temperature

Environmental factors were automatically measured and stored in the computer. The data of outside air temperature, radiation, and greenhouse air temperature were recorded every half an hour.

### 4.3. Measurements of Leaf Gas Exchange Parameters

A portable photosynthesis system (CIRAS-3, PP Systems, Amesbury, MA,103 USA) equipped with the leaf chamber fluorometer (PLC3 Universal Leaf Cuvette, 18 × 25 mm window, CFM-3) was used to measure leaf gas exchange parameters, including the net photosynthetic rate (*P*_n_), stomatal conductance (*G*_s_), intercellular CO_2_ concentration (*C*_i_), and transpiration rate (*T*_r_). The level of irradiance for measurements was set at 1000 µmol photons m^−2^s^−1^. The air temperature, CO_2_ concentration and relative humidity used were those of the greenhouse conditions. The above and middle fully developed leaves of different treatments were acclimated to the irradiance level approximately 2 min before recording. The measurements were conducted at approximately 10–12 am 8 days before treatment and 4 days, 16 days, 54 days, and 84 days after treatment.

### 4.4. Measurements of Leaf Relative Chlorophyll Content

The leaf relative chlorophyll contents were measured on the same days as the photosynthesis measurements using a chlorophyll meter (SPAD 502, Minolta, Japan). The measurements were made on the intact top and middle fully developed leaves, and each treatment measurement was replicated at least five times [27].

### 4.5. Measurements of Soluble Protein, Fibre, Total Phenolic, Flavonoids, Nitrate and Nitrite Content

Cucumber fruit quality parameters were measured 4 days, 16 days, 54 days, and 84 days after treatment. Soluble protein content was measured using Coomassie brilliant blue G-250 staining, which used the method of Bradford [28]. Fiber content was measured referencing the improved method of Śmiechowska and Dmowski [29]. Total phenolic, and flavonoids were determined by test kit (Suzhou Comin Biotechnology CO., Ltd., Suzhou, China) according to Dewanto et al. [30].

To measure nitrate and nitrite content, fresh fruit tissues (2 g) received from homogenates by Mincer were added to a test tube with 10 mL distilled water. The test tube was heated in boiling water for 30 min and then cooled down with tap water. The extract in the tube was filtered into a 100 mL flask, and the residue was repeatedly washed. Finally, distilled water was added to the flask to make the solution of 100 mL. The filtrate was used for nitrate and nitrite content determinations. The nitrate concentration of the filtrate was detected using the method of Cataldo et al. [31]. The content of the nitrite in the filtrate was determined by the method of Kaur et al. [32].

### 4.6. Measurements of Leaf Mineral Element Contents

Fully developed leaves for the upper and middle of different treatments were randomly selected after 16 days, 36 days, 54 days, and 84 days of treatment heated at 105 °C for 2 h and then dried at 80 °C in an oven for 3 days. The leaf mineral element contents were tested following the advice of Eurofins (branch company of Suzhou, China), and the method described by Song et al. [33] with modifications. The total nitrogen (TN) was analyzed using the near-infrared method. Molybdenum (Mo) was determined using inductively coupled plasma mass spectrometry (ICP-MS). The contents of chloride (Cl), phosphorus (P), potassium (K), calcium (Ca), magnesium (Mg), zinc (Zn), sulfur (S), iron (Fe), manganese (Mn), boron (B), and copper (Cu) were determined by flow analysis using inductively coupled plasma atomic emission spectrometry (ICP-AES).

### 4.7. Statistical Analysis

Analysis of variance (ANOVA) was conducted using SAS v. 9.3 (SAS Institute Inc., Cary, NC, USA). Each value is presented as the mean ± standard deviation (SD) with a minimum of three replicates. Differences between the treatment means were tested using the least significant difference (LSD) method at α = 0.05 level of significance. The figures were plotted using Origin 8.5 (OriginLab, Northampton, MA, USA).

## 5. Conclusions

This study demonstrated that it is possible and environmentally friendly to use a zero discharge method of nutrients to the environment in hydroponic cucumber production in a greenhouse. The zero discharge treatment did not change cucumber growth parameters, photosynthesis, relative chlorophyll content, cucumber yield, and fruit quality. As leaf mineral element contents decreased, leaf trace elements of Fe, Mn, Cu, and Mo can be appropriately supplied on treatment days. Growers need to pay more attention to plant adaptation at earlier stages of the zero discharge treatment, and the management of irrigation, plants, and climate control. A cultivation method with zero discharge of nutrient solution can reduce the energy costs of disinfection, save water and fertilizers, and reduce the pollution to the environment in cucumber cultivation.

## Figures and Tables

**Table 1:** The averaged leaf mineral element contents in the two irrigation treatments over the four measurements.

Treatments	Leaf Mineral Elements						
	Macro minerals (mmol·kg^−1^ DW)	N	P	K	Ca	Mg	S
Control		4255.5 ± 64.5 a	271.1 ± 7.8 b	891.9 ± 22.3 b	1334.7 ± 38.9 a	334.4 ± 7.9 b	249.3 ± 2.6 b
Zero discharge		4268.7 ± 88.3 a	314.1 ± 2.8 a	1015.6 ± 4.6 a	1390.5 ± 60.9 a	399.0 ± 7.7 a	288.4 ± 1.3 a
	Trace elements (µmol·kg^−1^ DW)	Fe	Mn	Zn	B	Cu	Mo
Control		3321.2 ± 277.0 a	4294.4 ± 188.7 a	1561.8 ± 23.9 b	8896.3 ± 331.2 a	135.4 ± 2.8 a	5.0 ± 0.4 a
Zero discharge		1868.8 ± 31.6 b	3363.8 ± 44.5 b	2268.8 ± 52.2 a	8878.8 ± 16.1 a	92.1 ± 2.2 b	2.5 ± 0.2 b

Data represent the mean ± SD (*n* = 3). Different letters indicate significant differences at α = 0.05 based on the least significant difference (LSD) test.

**Table 2:** Element components in the mother nutrient solutions A and B in different tanks.

A	kg/1000L	A	g/1000 L	B	kg/1000 L
Ca(NO_3_)_2_·4H_2_O	175	MnSO_4_·H_2_O	180	KNO_3_	50
EDTA-Fe (13%Fe)	1.5	ZnSO_4_·7H_2_O	125	KH_2_PO_4_	25
		Na_2_B_4_O_7_·10H_2_O	280	K_2_SO_4_	10
		CuSO_4_·5H_2_O	25	MgSO_4_·7H_2_O	60
		Na_2_MoO_4_·2H_2_O	15		

## Data Availability

All data used during the study are available from the author Xiaotao Ding by request (e-mail: xiaotao198108@163.com).
